# Neuroimaging correlates of handwriting quality as children learn to read and write

**DOI:** 10.3389/fnhum.2014.00155

**Published:** 2014-03-19

**Authors:** Paul Gimenez, Nicolle Bugescu, Jessica M. Black, Roeland Hancock, Kenneth Pugh, Masanori Nagamine, Emily Kutner, Paul Mazaika, Robert Hendren, Bruce D. McCandliss, Fumiko Hoeft

**Affiliations:** ^1^Division of Child and Adolescent Psychiatry, Department of Psychiatry, UCSFSan Francisco, CA, USA; ^2^Department of Psychology, Pacific Graduate School of Psychology, Palo Alto UniversityPalo Alto, CA, USA; ^3^Graduate School of Social Work, Boston CollegeChestnut Hill, MA, USA; ^4^Haskins Laboratories, Yale UniversityNew Haven, CT, USA; ^5^Division of Behavioral Sciences, National Defense Medical College Research InstituteTokorozawa, Saitama, Japan; ^6^Department of Psychiatry and Behavioral Sciences, Center for Interdisciplinary Brain Sciences Research, Stanford University School of MedicineStanford, CA, USA; ^7^Department of Psychology and Human Development, Vanderbilt UniversityNashville, TN, USA; ^8^Department of Neuropsychiatry, Keio University School of MedicineShinjuku-ku, Tokyo, Japan

**Keywords:** phonological processing, voxel-based morphometry, functional MRI, inferior frontal gyrus pars triangularis, writing, reading

## Abstract

Reading and writing are related but separable processes that are crucial skills to possess in modern society. The neurobiological basis of reading acquisition and development, which critically depends on phonological processing, and to a lesser degree, beginning writing as it relates to letter perception, are increasingly being understood. Yet direct relationships between writing and reading development, in particular, with phonological processing is not well understood. The main goal of the current preliminary study was to examine individual differences in neurofunctional and neuroanatomical patterns associated with handwriting in beginning writers/readers. In 46 5–6 year-old beginning readers/writers, ratings of handwriting quality, were rank-ordered from best to worst and correlated with brain activation patterns during a phonological task using functional MRI, and with regional gray matter volume from structural T1 MRI. Results showed that better handwriting was associated negatively with activation and positively with gray matter volume in an overlapping region of the pars triangularis of right inferior frontal gyrus. This region, in particular in the left hemisphere in adults and more bilaterally in young children, is known to be important for decoding, phonological processing, and subvocal rehearsal. We interpret the dissociation in the directionality of the association in functional activation and morphometric properties in the right inferior frontal gyrus in terms of neural efficiency, and suggest future studies that interrogate the relationship between the neural mechanisms underlying reading and writing development.

## Introduction

Writing by hand occupies 30–60% of a child's school day (Stewart, 1992; Simner, 1998; Feder and Majnemer, 2007; Sassoon, 2007) and correlates with self-esteem and future academic success. Children with deficient handwriting (10–30% of children; Karlsdottir and Stefansson, 2002) take longer to complete writing tasks such as homework, which can increase the difficulty of schoolwork and result in oppositional attitudes toward writing assignments that can generate problems both at school and at home (Racine et al., 2008). Crucially, handwriting performance also shares links with other language related skills. Of particular relevance, there are important associations between reading and learning to write. Studies have shown that learning to write can improve letter perception (Longcamp et al., 2005), pseudoletter learning (Richards et al., 2011), and word reading (e.g., Berninger et al., 2004, 2006a; James and Engelhardt, 2012). Correspondingly, children with learning disabilities such as developmental dyslexia, a specific reading impairment that is believed to have phonological deficits at its core, often display writing difficulties (O'Hare and Khalid, 2002).

With the increasing integration of computers into the education system, the implied implications of reduced handwriting practice have strengthened the interest of scientific investigators. Recent neuroimaging studies have concluded that while free-form handwriting practice clearly supports reading acquisition, typing (Longcamp et al., 2005) and even tracing (James and Engelhardt, 2012) do not. Impressively, James and Engelhardt (2012) showed that preliterate children recruit well established reading related brain regions, such as the fusiform gyrus, posterior parietal cortex, and the inferior frontal gyrus, during letter processing exclusively after handwriting practice compared to typing or tracing. The emerging consensus is that the motor experience of manually creating letterforms helps children discriminate the essential properties of each letter, which leads to more accurate representations bolstering both skilled letter recognition and later reading fluency. Therefore, understanding the underlying neurological mechanisms that support handwriting development is important not only for its independent relevance to educational achievement, but also for its supportive role in successful acquisition of other language skills such as reading.

The neurological basis underlying handwriting is not well understood but converging evidence points to key regions including: (a) the fusiform gyrus, which has apparent selectivity to letter (James and Gauthier, 2006) and word stimuli (Cohen et al., 2000; Cohen and Dehaene, 2004) over other visual stimuli and may provide a perceptual component for deriving “word-form” representations that facilitate grapheme writing (Dehaene et al., 2005; James, 2010); (b) the superior parietal lobule (SPL), a region important for carrying out actions in space (Goodale and Milner, 2005) that is thought to be involved in both visuospatial and visuomotor processing (Petrides and Pandya, 1984; Morecraft et al., 2004; Segal and Petrides, 2012), and the execution of writing sequences (Otsuki et al., 1999); (c) the inferior frontal gyrus (IFG), implicated for its involvement in phonological processes (Eckert et al., 2003) and its associations with encoding letterforms and words (Grafton et al., 1997; Berninger and Winn, 2006b; Longcamp et al., 2008); and (d) Exner's area, thought to be the interface of orthographic or graphemic representations and the complex movement sequences necessary for generating letters and words (Anderson et al., 1990; Lubrano et al., 2004; Roux et al., 2009) and may also be involved in retrieving letter shapes from memory (James and Gauthier, 2006).

While advances have been made, a complete understanding of the brain's writing system remains elusive. The inherent complexities involved in the task of writing, coupled with the excessive variability of its definition in the existing literature, make it challenging to delineate the extent of neuronal specialization specific to handwriting from other inter-related aspects, such as spelling. In a recent neuroimaging metaanalysis of writing in adults, however, authors dissociated linguistic input and motoric aspects of writing and identified IFG for processing linguistic input as it relates to writing, and left superior frontal sulcus/middle frontal gyrus (Exner's), left superior parietal lobule, and the right cerebellum as “writing-specific” regions (Planton et al., 2013). Another study has shown that the brain differentially recruits specialized regions based on a multiplicity of letter representations (e.g., motoric similarities “B” vs. “P,” visual similarities “A” vs. “R,” abstract similarities “A” vs. “a”) (Rothlein and Rapp, 2014). What is lacking is detailed examination of the emergence of “neural specialization” during the period when writing skills develop and the brain basis of the underlying process (except see work by Karen James cited in this paper). Further, more investigations of association between the brain basis of writing and other processes of written language such as reading is greatly needed. Findings from such studies may not only offer important insights to improve research methodology and educational instruction, but may also contribute to a fuller understanding of the development of written language processing in the human brain.

The present study sought to focus on the neural correlates of *handwriting quality* in children at the beginning of formal handwriting instruction. Operationally, handwriting quality refers to the legibility, form, slant, spacing, and general appearance of letters and words. Handwriting researchers have generally agreed on the relevance of these key features (Freeman, 1959; Kaminsky and Powers, 1981; Graham, 1982; Ziviani and Elkins, 1984; Graham and Weintraub, 1996). Given that handwriting exposure in preliterate children has been associated with reading related processes such as letter perception and related brain activation (James and Engelhardt, 2012), it is plausible to consider that especially during early stages of development, handwriting also share links with phonological processing a skill that for decades has been casually linked to reading acquisition (Castles and Coltheart, 2004; Byrne et al., 2008). Therefore, we sought to investigate the unproven idea that handwriting and reading may rely on a common neuroanatomical mechanism at an early developmental stage of reading/writing. We therefore hypothesized handwriting quality will be associated with neuroanatomical patterns in one or more of the following: (a) IFG if phonological decoding coding is relevant to handwriting quality, (b) Exner's area if successful integration of orthography and motor programs are relevant to handwriting quality, (c) SPL if sequential motor movements and/or kinesthetic modulation are relevant to handwriting quality, and/or (d) fusiform gyrus if visual letter or word recognition is relevant to handwriting skill. Then, in order to investigate the direct relationship of handwriting and reading abilities, in particular of phonological processing, we associated handwriting quality with brain activation during a task aimed at engaging the brain's phonological processing circuit. If handwriting is associated with the development of reading, and phonological processing plays a causal and reciprocal relationship with reading acquisition, we hypothesized that brain activation patterns associated with phonological processing, may also be associated with handwriting skills in these emergent readers / writers.

## Materials and methods

Our data come from a study focused on examining brain activation during phonological processing and the relationship between reading-related behavioral measures. While the original study was not focused on the brain basis of handwriting, and hence the behavioral measures and fMRI tasks were not necessarily optimized for the purpose of the current study, yet these data provided an opportunity to investigate whether neuroanatomical patterns and brain activation during phonological processing are associated with handwriting in beginning readers and writers.

### Participants

A total of 51 (29 boys, 22 girls) healthy, native English-speaking 5- and 6-year-old children (aged 5.59 ± 0.42) toward the beginning of formal schooling participated in this study. Standard behavioral assessments of the children, along with MRI data were collected. We later excluded five left-handed children, leaving 46 remaining right-handed children to be included in all analyses unless there was missing data or excessive movement motion or severe scanner artifacts (fMRI analyses, *N* = 41). While we did not exclude children based on attention deficit hyperactivity disorder (ADHD) for example, the children in this study did not have any parental report of formal diagnosis of neurological or psychiatric disorders besides specific learning disabilities; they were not on medication and had no contraindications to MRI. Behavioral Assessment System for Children −2 (Reynolds and Kamphaus, 2006) showed that all children were within typical range.

To help prepare participants for imaging, parents received a packet of informational material, including a CD of common scanner sounds and a DVD of a child going into the scanner. Parents were instructed to review these supplemental materials with their children to familiarize and desensitize participants to the scanner environment. In addition, children participated in a guided MRI simulation at the center where they practiced lying still in the bore and underwent training to minimize motion related artifacts. Participants with excessive, uncorrectable motion were eliminated from the study.

The Stanford University Panel on Human Subjects in Medical Research and the University of California, San Francisco Human Research Protection Program approved the study and informed consent and assent were obtained from parents/guardians and participants, respectively.

### Behavioral measures

We administered a standard battery of neuropsychological assessments, which included the Woodcock-Johnson III (WJ-III) Spelling (Woodcock et al., 2001), an untimed real-word spelling test, in order to assess spelling accuracy and handwriting quality (see below); the Beery Visual-Motor Integration (BVMI; Beery and Beery, 2004), where children copied and traced a series of moderately complex geometric figures; and the Oromotor Sequences subtest from the Developmental Neuropsychological Assessment (NEPSY-II; Korkman et al., 2007) to assess oral-motor praxis, or the ability to sequence oral-motor movements without articulation difficulty, without visual demands. Additionally, the Home Literacy Inventory (Marvin and Ogden, 2002) was used to investigate the differences in the exposure and practice of reading activities at home.

### Handwriting quality

In order ensure participants were unaware that their handwriting was under investigation, handwriting samples were drawn from the WJ-III Spelling subtest were used as a basis for assessing and defining handwriting skills. Two blinded investigators, who were trained to score handwriting quality holistically based on letterform, slant, spacing and general appearance irrespective of spelling errors and speed, each rank-ordered (1 = poor handwriting, 51 = best handwriting) participants' writing sample from best to worst three times. Since spelling inaccuracies can inadvertently bias rankings, writing samples included both letters and small words. Intraclass correlation coefficients were calculated to examine intra-rater reliability (Cronbach's alpha = 0.994 for rater 1; 0.989 for rater 2), and inter-rater reliability (Cronbach's alpha = 0.980) was calculated after the three sets of scores were averaged across raters. The final ranking used was based on the mean of each investigator's scores.

### Visuomotor (copying) skills

A subset of test items (items 17–19) from the BMVI task was selected by the investigators to evaluate visuomotor skills; these items were developmentally appropriate, yet were also sufficiently difficult. Specifically, these were the most difficult items (non-letter objects) that all participants were able to complete. According to the manual, the validity and reliability of the task are sufficient for the age of our participants (Beery, 1997). Following the same rank-ordering procedures as for handwriting quality, two blind investigators rated participants' reproductions, which were based on copying geometric shapes (1 = poor reproduction, 51 = best reproduction). Intraclass correlation coefficients were calculated to examine intra-rater reliability (Cronbach's alpha = 0.993 for rater 1; 0.974 for rater 2) and inter-rater reliability (Cronbach's alpha = 0.969) was calculated after the three sets of scores were averaged across raters. The final ranking used was based on the mean of each investigator's scores.

### Functional MRI tasks

**Figure 1:**
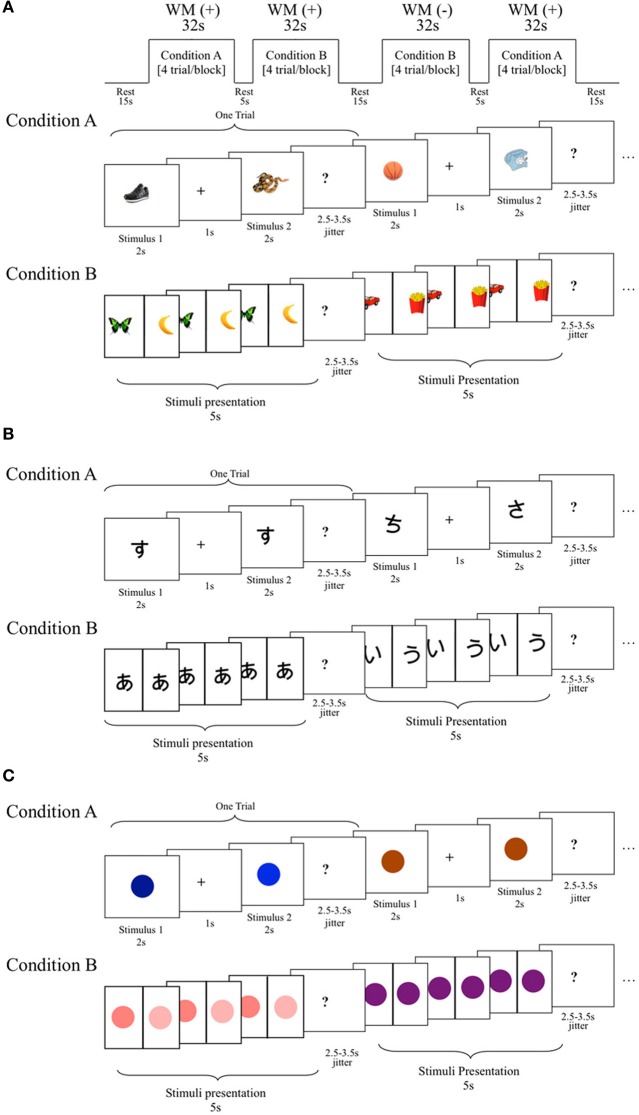
**Schematic diagram of the functional magnetic resonance imaging (fMRI) tasks. (A)** Phonological processing fMRI task. In this block design fMRI task, participants were asked to determine whether the name of the pictured stimuli begin with matching sounds. Condition A: required working memory (WM+), stimulus presented one after another for 2 s each with 1 s intervals (must be retained across a delay). Condition B: no working memory required (WM−), stimuli presented side-by-side for 5 s. Conditioned collapsed for the purposes of this study. **(B)** A visual-symbol matching block design fMRI task. Design was the same as phonological task except that Japanese hiragana symbols were presented instead of pictured objects, and participants were asked to determine whether these unrecognized symbols matched. Note: working memory was pertinent to the parent study, but it was not crucial to this study, and for the purposes of this study WM+ and WM− conditions were collapsed into one condition. **(C)** A color discrimination block design fMRI task. Design was the same as the other tasks except that colors were presented instead of pictured objects or symbols, and participants were asked to determine whether these colors were the same. Note: working memory was pertinent to the parent study, but it was not crucial to this study, and for the purposes of this study WM+ and WM− conditions were collapsed into one condition.

Three tasks measuring a range of cognitive abilities were used to investigate neurological associations to handwriting. The first was a phonological processing task in which participants were asked to determine if the first sound of the names of two pictures of common objects matched (Figure 1A). This task was adapted from a sound-matching subtask of the Comprehensive Test of Phonological Processing (Wagner et al., 1999) and is well established as reliable in phonological processing investigation (e.g., Katzir et al., 2005). The second task was a non-verbalizable visual-symbol matching task in which participants were presented with unfamiliar Japanese hiragana (no participants knew that they were letters from another language). Visually similar hiraganas (e.g., 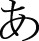 vs. 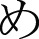) were presented to try to maximize difficulty (Figure 1B). This task was used to at least partially account for visual input and motor response often associated with fMRI tasks that requires processing of letters and explicit motor responses (Henson et al., 2000). Finally, the third task was a color-matching task in which participants were asked to determine whether two colors matched (Figure 1C). The pair of stimuli were of the same hue but of different lightness with close value optimized in a pilot study to avoid using names of the colors to perform the task and to maximize difficulty. Although there is no assumed relationship between color-matching and handwriting, this task was included as another task to help account for some of the confounds, such as the color dissimilarities in the stimuli used in the phonological task and the decision making nature of all three tasks. These latter two tasks were only obtained in a portion of the children (*N* = 18). We therefore performed a secondary analysis of the phonological fMRI task matched to include only those participants that also completed the visual-symbol matching and color matching control tasks when comparing between tasks. The results of the phonological fMRI task were unchanged regardless of the sample-size and were specific to the phonological task.

All three tasks utilized the same procedure. Each required participants to determine whether two visually presented stimuli matched for either the first syllables of the names of pictures, visual symbols or color. Stimuli were presented simultaneously in one condition (without enabling working memory, WM−) and after a small delay (enabling working memory, WM+) (Figures 1A–C). In this study, we report results collapsing the two conditions. The exploration of the role of working memory in reading and writing may answer important theoretical questions and should be examined in future studies. This is not however, explored further in the current study because of the non-significant difference between conditions, which may have been due to a number of factors such as the short interstimulus interval. Rest was used as the control condition because our preliminary study showed difficulty of children performing a phonological task (sound matching of first syllable) alternating with a control condition (such as visual shape matching). We therefore opted to use the two other visual fMRI tasks to show specificity of the effects to the sound-matching task. Participants completed two runs of each task. Each of the two runs began with a 6 s countdown and a 2 s rest period. In the WM− condition, stimuli were presented side-by-side continuously for 3.5 s (followed by a 2.5–3.5 s jitter with a mean average of 3 s), whereas the WM+ condition displayed stimuli at the center one at a time for 2 s each with a jitter of 2.5–3.5 s (mean average 3 s) between stimuli (paired stimuli were also followed by a 2.5–3.5 s jitter with an mean average of 3 s). There were 5 trials per block. The 4 task blocks in each run were 32 s in duration and the order of the condition was varied from Run1 (WM −→WM+→WM+→WM−) and Run2 (WM+→ WM −→ WM −→WM+), with a 5, 15, and 5 s intervals between blocks. Participants (*N* = 41) completed 2 runs, with each run being 170 s in length (174 s total with the first 4 s of the scans in each run being discarded to establish equilibrium in MR signal). All stimuli were presented against a plain, white background and participants responded with their right finger if the stimuli matched and with their left finger if they did not match. Since participants may think of different words than intended for the pictured stimuli used in the phonological task, *post-hoc* testing asking names of each picture was performed for each child to verify whether there were discrepancies between potentially ambiguous images that may have alternative, yet still correct, pronunciations. This was necessary to ensure accurate task performance calculation tailored for each subject. Due to the young age of participants, data were used if their task accuracy total was greater than chance. Overall accuracy as well as reaction times for all correctly answered trials are shown in Table 1.

**Table 1:** **Demographics and correlations**.

**Measure**	**Association with handwriting quality**					
	**Mean (*SD*)**	***t, r*, or *p***	***p*-value**			
Age	5.59 (0.42)	*r* = 0.27	0.075			
Gender	26 boys / 20 girls	*t* = 2.64	0.012^*^			
Handedness (all right)	82.27(19.81)	*r* = 0.17	0.26			
Mother's education (years)	17.02 (2.07)	*P* = −0.12	0.42			
	**Raw scores**			**Standard/scaled scores**		
	**Mean (*SD*)**	***r***	***p*−value**	**Mean (*SD*)**	***r***	***p*−value**
Handwriting quality^a^	0 (1)	1	0.00^**^	NA	NA	NA
Phonological fMRI (accuracy)	72.01% (12.66)	−0.137	0.447	NA	NA	NA
Phonological fMRI (reaction time)	2601.78 ms (511.56)	−0.115	0.462	NA	NA	NA
FSIQ^b^ WJIII BIA^c^	NA	NA	NA	119.24 (2.07)	−0.17	0.27
PPVT^d^	119.57 (14.11)	0.016	0.92	122.00 (9.21)	0.20	0.18
WRMT^e^ letter identification	31.48 (7.96)	0.13	0.39	111.87 (10.95)	0.20	0.18
WRMT word identification	15.26 (19.38)	0.11	0.46	119.41 (31.84)	0.090	0.55
CTOPP^f^ phonological awareness	NA	NA	NA	112.54 (14.78)	0.040	0.79
CTOPP elision	6.59 (4.44)	−0.099	0.51	12.00 (2.87)	−0.099	0.51
CTOPP blending	8.45 (3.43)	−0.13	0.38	12.48 (2.04)	−0.154	0.31
CTOPP phonological memory	NA	NA	NA	106.28 (11,75)	0.090	0.21
RAN^g^ object	1.76 (1.40)	0.097	0.52	101.93 (15.41)	0.030	0.84
RAN color	0.39 (1.02)	0.079	0.60	99.43 (15.53)	−0.050	0.75
Visuomotor (BVMI^h^ rank)	0 (1)	0.45	0.0020^**^	NA	NA	NA
WJIII spelling	14.48 (3.55)	0.36	0.013^*^	112.04 (12.13)	0.18	0.24
BVMI right	16.43 (2.75)	0.38	0.010^**^	108.39 (14.53)	0.25	0.092
NEPSY^i^ oromotor	38.5 (10.40)	−0.0050	0.96	3.00 (0.84)	0.090	0.56
Home literacy inventory	8.62 (3.47)	0.036	0.815	NA	NA	NA
TGMV^j^	710.95 (63.24)	−0.14	0.36	NA	NA	NA
TWMV^k^	455.7 (43.97)	−0.17	0.27	NA	NA	NA

* *p < 0.05 level (2-tailed)*.

** *p < 0.01 level (2-tailed)*.

a *Writing samples derived from Woodcock-Johnson III Spelling (subtest from Test of Cognitive Abilities)*.

b *Full Scale Intelligence Quotient*.

c *Brief Intellectual Ability*.

d *Peabody Picture Vocabulary Test*.

e *Woodcock Reading Mastery Tests*.

f *Comprehensive Test of Phonological Processing (Phonological Awareness = Elison + Blending)*.

g *Rapid Automatized Naming*.

h *Beery-Buktenica Developmental Test of Visual-Motor integration*.

i *Developmental Neuropsychological Assessment*.

j *Total Gray Matter Volume*.

k *Total While Matter Volume*.

### Structural and functional MRI data acquisition

Imaging was conducted at the Lucas Center for Imaging at the Stanford University School of Medicine. Imaging data was acquired using GE Healthcare 3.0 Tesla 750 scanner and an 8-channel phased array head coil (GE Healthcare, Waukesha, WI). Images acquired included an axial-oblique 3D T1-weighted sequence (fast spoiled gradient recalled echo [FSPGR] pulse sequence, inversion recovery preparation pulse [TI] = 400 ms; repetition time [TR] = 8.5 ms; echo-time [TE] = 3.4 ms; flip angle = 15°; Receiver bandwidth ± 32 kHz; slice thickness = 1.2 mm; 0.86 × 0.86 mm in-plane resolution; 128 slices; number of excitations = 1; field-of-view [FOV] = 22 cm; acquisition matrix = 256 × 192). The total scan time was 4:54.

Functional MRI (fMRI) data were acquired using an axial 2D GRE Spiral In/Out (SPRLIO; Glover and Law, 2001) pulse sequence (*TR* = 2000 ms; *TE* = 30 ms; flip angle = 80°; Receiver bandwidth +125 kHz; slice thickness = 4.0 mm; number of slices = 31, descending; 3.44 × 3.44 mm in-plane resolution; number of temporal frames = 85; FOV = 22 cm). The total duration of each task was 5:12.

### Regions of interests (ROIs)

Bilateral regions-of-interest (ROIs) used in this study were: (a) pars triangularis and pars opercularis of the IFG (IFGtri and IFGop, respectively) based on previous studies of language development, literacy, and handwriting in IFG (Longcamp et al., 2003, 2008), (b) Exner's region based on its role in generating graphemic-motor commands (Exner, 1881; Ritaccio et al., 1992; Roux et al., 2010; Planton et al., 2013), (c) SPL based on its involvement with complex motor sequences that contribute to the accuracy of written expression (Alexander et al., 1992; Sakurai et al., 2007), and (d) fusiform gyrus based on its role in letter (James and Gauthier, 2006) and word processing (Cohen et al., 2000). Automated Anatomical Labeling (AAL) (Tzourio-Mazoyer et al., 2002) in the WFU PickAtlas toolbox (Maldjian et al., 2003) was used to generate ROIs (a), (c), and (d). Exner's area ROI (b) was selected based on a neuroimaging study (Matsuo et al., 2003) as a region of the left precentral gyrus (PreCG, BA 6), adjacent to BA 9 and BA 44 (Talairach coordinates [TAL]: −46, 3, 27). A sphere with a diameter of 10 mm centered around these coordinates was used as the Exner's area ROI.

### Preprocessing of fMRI images

Processing of fMRI data was performed with statistical parametric mapping software (SPM8; Wellcome Department of Cognitive Neurology, London, UK) in the MATLAB computing environment (The MathWorks, Natick, MA). After image reconstruction, each participant's data were slice time corrected, realigned to a reference volume and corrected for motion and artifacts using both SPM and in-house tools (http://www.nitrc.org/projects/art_repair/). Data were spatially normalized to Montreal Neurological Institute (MNI) space using normalization parameters obtained from the children's segmented gray matter images of high resolution T1 MRI normalized to standard template and applied to the mean functional image. Resultant images were resampled to 2 × 2 × 2 mm voxels in MNI stereotaxic space. Spatial smoothing was done with an 8-mm isotropic Gaussian kernel. Each participant's data were high pass filtered at 128 s, and analyzed using a fixed effects model examining task; rest was not modeled and was included as implicit baseline. Five of the 46 participant's data were not included (final *N* = 41) because of excessive motion (criteria: relative motion <1.0 mm), at or below chance task performance (criteria: accuracy ≤50%), and/or scanner artifact (*N* = 5).

### Statistical analyses of fMRI data: main analyses of interest

Statistical analysis was performed first using a fixed effects analysis for each participant modeling each condition. Task vs. rest contrasts were used for further group analysis for the purposes of this study as stated in the Functional MRI Tasks section above. Using random effects analysis, a one sample *t*-test was performed to examine brain regions that were active during the phonological fMRI task [*p* = 0.05 family-wise error (FWE) corrected, at the whole brain level].

Next, simple correlation analysis was performed between brain activation during the fMRI tasks and handwriting skills in the ROIs using a statistical threshold of *p* = 0.05 family-wise error (FWE) corrected for height using small volume correction. We also examined voxel-by-voxel associations in the whole brain at a more lenient threshold of *p* = 0.001 uncorrected for height to examine whether there are any clusters outside the ROIs that showed significant effects at this more lenient threshold.

### Statistical analyses of fMRI data: control analyses

Control analyses were performed in several ways. First, analyses examining associations between handwriting quality and brain activation during the phonological task regressing out the non-handwriting motor and writing abilities such as visuomotor skills (rank order of BVMI), oromotor skills (NEPSY-II oromotor subtest), and spelling (WJ-III spelling subtest), as well as correlated demographic variables [age (there was a trend for significant effects of older age correlating with better handwriting), gender (males had significantly poorer handwriting than females)] were performed. Second, ROI based regression analyses between brain activation during the phonological task and these aforementioned non-handwriting motor and writing abilities were performed. Statistical threshold was set similarly to the main analysis at *p* = 0.05 FWE corrected for the ROIs (and *p* = 0.001 uncorrected for the whole brain to examine whether there are any clusters outside the ROIs that showed significant effects at this more lenient threshold). Third, whole-brain and ROI analyses were performed correlating brain activation during the supplemental visual-symbol matching and color matching tasks and handwriting skills (rank order of WJ-III spelling writing samples). Since we only had data from these tasks in half of the participants, in order to show that the significant effect in the meta-phonological task and not the supplementary tasks was not due to power issues, we went back and repeated the main correlation analysis (between brain activation during the meta-phonological processing task and handwriting skills) using a smaller sample with data from both the meta-phonological and supplementary tasks.

### Preprocessing and statistical analysis of T1 structural MR images

Voxel-based morphometry (VBM) analysis of T1-weighted MRIs was performed using Statistical Parametric Mapping, version 8, (SPM8) (http://www.fil.ion.ucl.ac.uk/spm). After alignment to AC-PC axis, T1-weighted images were bias-corrected and segmented to gray matter, white matter, and cerebrospinal fluid, using SPM8 default tissue probability maps and “New Segment” tool, which also included an affine regularization to warp images to the included International Consortium for Brain Mapping (ICBM) template, producing rigidly aligned tissue class images. Inter-subject registration was achieved with Diffeomorphic Anatomical Registration Through Exponentiated Lie Algebra (DARTEL), using default settings. Jacobian-scaled (“modulated”), warped tissue class images were created with DARTEL's “Normalize to MNI Space” tool, which spatially normalized images to MNI space, converted voxel sizes to 1.5 × 1.5 × 1.5 mm^3^ to match the DARTEL template, and smoothed images with a standard Gaussian filter of full-width at half-maximum (FWHM) equal to 8 mm. For each participant, segmentation and normalization accuracy were manually inspected. 41 of 46 participants were included in this analysis due to usability issues caused either by artifacts or excessive motion. Statistical analyses were performed similarly to fMRI analyses using the same statistical thresholds but additionally controlling for total gray matter volume. Finally, associations between regional gray matter volume and brain activation were performed where the spatial location at least partially overlapped. The reported Talairach coordinates were converted from MNI space using the mni2tal function (http://www.mrc-cbu.cam.ac.uk/Imaging/Common/mnispace.shtml). Talairach Daemon (Research Imaging Center, University of Texas Health Science Center; Lancaster et al., 1997, 2000) and the atlas by Talairach and Tournoux (1988) were initially used to identify Brodmann Areas. The final anatomic locations are reported according to their anatomic location overlaid on the custom template.

## Results

### Behavioral results

Table 1 shows demographic and behavioral characteristics as well as associations between these measures and handwriting quality. Age, handedness, and maternal education, often used as a proxy for environment, did not yield any significant associations with handwriting performance (all *p*'s > 0.05). However, as one might expect based on the fact that the handwriting measure was ranked-ordered and not standardized, even though the range of ages in these children were narrow (5–6 years of age), age showed a trend for significant positive association with handwriting [*r*_(44)_ = 0.27; *p* = 0.075], and gender effects were found [*t*_(44)_ = 2.64, *p* = 0.012] with boys demonstrating significantly weaker handwriting performance as compared to girls. Further, while handwriting performance was not significantly correlated with spelling standard scores [Table 1, *r*_(44)_ = 0.18, *p* = 0.24], spelling raw scores were significantly related [*r*_(44)_ = 0.36, *p* = 0.013]. (Since the ranking of handwriting quality was not a standardized measure, this was expected). Visuomotor skill ratings (see above for definition) were also significantly correlated with BVMI standard scores, which is expected since visuomotor integration skill was the construct being evaluated [*r*_(44)_ = 0.658, *p* < 0.001]. We also found, as anticipated, that rater's ranking of handwriting and visuomotor skills were associated with one another [*r*_(44)_ = 0.45, *p* = 0.002].

### fMRI results

**Figure 2:**
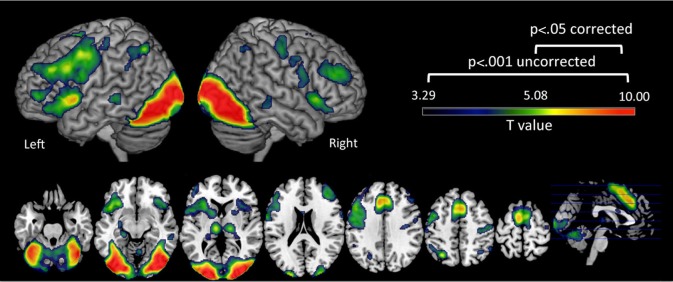
**Brain activation during the phonological processing fMRI task.** Clusters in warm colors indicate those significant at *p* < 0.05 family wise error (FWE) corrected. Those significant at *p* < 0.001 uncorrected, cluster extent = 10 are also included to show the extent of these clusters at a more lenient threshold. Note: Left Hemisphere is shown on left side.

First we examined brain regions that showed significant activation during the reading-related phonological processing task in all participants. We found that these emerging readers elicited significant activation at *p* = 0.05 corrected in bilateral (left > right) IFG, left superior, middle frontal gyrus and PreCG, left inferior parietal lobule and bilateral occipito-temporal region (Figure 2, Table 2). It is important to note that the behavioral profiles of participants included in this study are not representative of a normal population (see Table 1), so the results presented here are not yet generalizable.

**Table 2:** **Regional brain coordinates**.

**Brain region**	**Brodmann area (BA)**	**TAL coordinates**			***T* values (peak)**	***P* value (FWE)**	**Cluster size (voxels)**
		***x***	***y***	***z***			
Right fusiform, inferior, middle occipital gyri	18, 19	42	−78	−3	15.35	<0.001	11557
		38	−66	−8	15.31	<0.001	
		26	−93	10	13.6	<0.001	
Left medial frontal, right cingulate gyri	9, 6, 32	−8	27	30	7.92	<0.001	2125
		−8	1	59	7.74	<0.001	
		6	21	39	7.44	<0.001	
Left inferior frontal, superior temporal gyri, lentiform nucleus (Putamen)	47, 22	−28	21	−3	7.71	<0.001	798
		−46	11	−4	7.53	<0.001	
		−18	10	1	5.43	0.020	
Left parahippocampal gyrus	27	−20	−29	−2	6.77	0.001	88
Left superior parietal lobule	7	−30	−58	47	6.34	0.002	60
Left middle frontal gyrus	6, 9, 46	−46	6	42	6.21	0.002	424
		−50	19	27	5.96	0.005	
		−48	36	24	5.94	0.005	
Left thalamus		−10	−17	5	6.15	0.003	114
Right inferior frontal gyrus	47	32	27	−3	6.13	0.003	163
Right thalamus (ventral posterior lateral nucleus)		16	−17	6	5.67	0.011	39
Right thalamus		22	−27	0	5.4	0.022	7
Right middle frontal gyrus	10	40	40	18	5.24	0.033	8
Right culmen		4	−65	−10	5.24	0.034	8
Left precentral gyrus	6	−63	3	20	5.2	0.037	3
Right declive		6	−55	−14	5.19	0.039	6
Left inferior frontal gyrus	10	−44	47	−2	5.16	0.041	2
Left declive		−10	−59	−16	5.13	0.044	1
Right middle frontal gyrus	10	44	48	20	5.12	0.046	2
Left precentral gyrus	6	−46	−2	30	5.09	0.049	1

**Figure 3:**
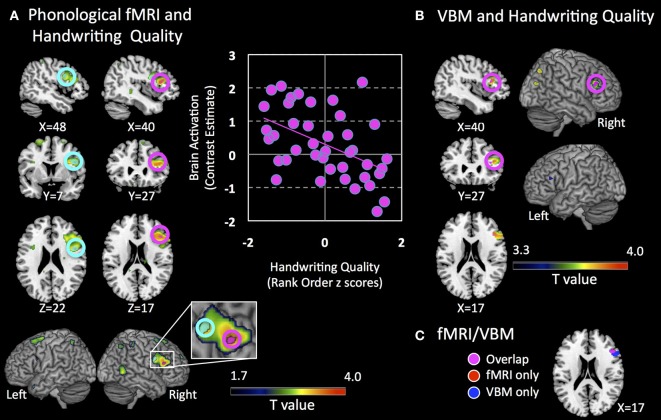
**Brain regions associated with handwriting quality. (A)** Clusters that show negative association with brain activation during a phonological processing fMRI task are shown. Pink circles indicate a cluster that is significant at *p* = 0.05 corrected (right inferior frontal gyrus pars triangularis, IFGtri), and cyan circles indicate a cluster that shows a trend *p* = 0.054 corrected (right Exner's area). Clusters indicate voxels significant at a lenient threshold of *p* = 0.05 uncorrected to show greater extent of activation. XYZ coordinates are in Talairach coordinates. Panel on the right shows a scatter plot representation of the cluster that shows significant negative association at *p* = 0.05 corrected (pink cluster). Brain activation is defined as contrast estimates, which are based on combined beta estimates of the phonological condition vs. rest. **(B)** Clusters that show positive association with regional gray matter volume are shown. Pink circles indicate clusters that are significant at *p* = 0.05 corrected in the right IFGtri. Clusters indicate all voxels significant at *p* = 0.001 uncorrected, cluster extent = 10 as reference to show the extent of these clusters at a more lenient threshold. A small cluster in the left IFGtri is observed at this threshold. XYZ coordinates are in Talairach coordinates. **(C)** Voxels that show overlap in fMRI activation from **(A)** and VBM gray matter volume from **(B)** in the right inferior frontal region. Note: Left Hemisphere is shown on left side.

Phonological activity was negatively associated with better handwriting quality in the right IFG within Broca's Area/ Brodmann Area 45 / pars triangularis [TAL: *X* = 44, *Y* = 24, *Z* = 15; peak *T* = 3.74; *p* = 0.033 corrected; mean cluster *r*_(39)_ = −0.43; Figure 3A]. Even when performing whole-brain analysis at a lenient threshold of *p* = 0.001 uncorrected, a cluster in the right IFGtri was the only region that showed a significant effect (TAL: *X* = 40, *Y* = 27, *Z* = 17; peak *T* = 3.77; *p* < 0.001 uncorrected). Exner's area (TAL: *X* = 48, *Y* = 7, *Z* = 22), although non-significant, also showed a distinctive trend in the same direction (*p* = 0.054 corrected). Given Exner's well-documented involvement in handwriting, this trend was included in Figure 3). No significant positive correlations were observed either at *p* = 0.05 corrected or *p* = 0.001 uncorrected. Activity in the right IFGtri cluster during the phonological task was also negatively correlated with CTOPP phonological memory composite scores (*r* = −0.31, *p* = 0.049) and memory for digits subtest (*r* = −0.37, *p* = 0.017).

Control analyses were performed in three ways. First, the negative correlation in the right IFGtri remained significant using whole-brain regression analysis of the phonological fMRI task even after regressing out variables that correlated with handwriting quality as well as other motor and writing skills such as age, gender, visuomotor skill (rank ordered BVMI responses), oromotor skills, BVMI (dominant/right hand) raw scores, and WJ-III Spelling raw scores (*r* = −0.369, *p* = 0.029).

**Figure 4:**
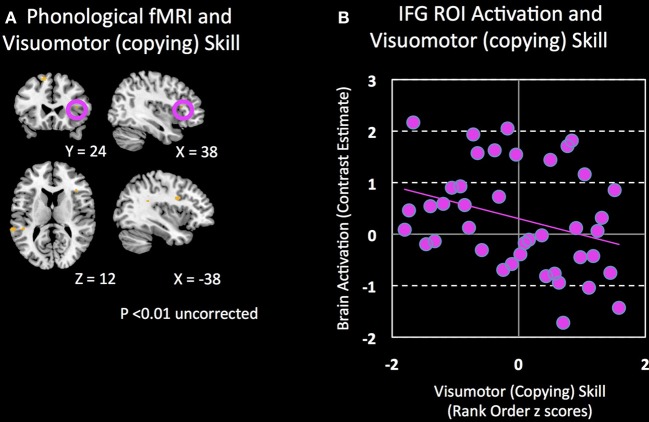
**Brain regions associated with visuomotor (copying) skill. (A)** Clusters that show a negative association (*p* = 0.01 uncorrected) between visuomotor (copying) skill and brain activation during a phonological processing fMRI task are shown. **(B)** Non-significant correlation between visuomotor (copying) skill and activation in the right IFG during phonological processing.

Second, control analyses were then performed using ROI-based (IFGtri and IFGop from AAL) and whole-brain regression between activation during phonological processing and motor and writing skills other than handwriting skills. Correlations between right IFG activation and unstandardized visuomotor skills (see Methods for definition) (peak *T* = 2.51; *p* = 0.19 corrected; *p* = 0.008 uncorrected; Figure 4), oromotor skills (peak *T* = 3.03; *p* = 0.071 corrected; *p* = 0.002 uncorrected) and spelling (peak *T* = 0.42; *p* = 0.85 corrected; *p* = 0.29 uncorrected) were not significant, controlling for age (either by regressing age out or by using normed scores).

Third, no significant positive or negative correlation was observed with handwriting quality and brain activation during either the visual-symbol matching or color matching tasks, demonstrating that the association is likely to be specific to the phonological processing task. Since we only had data from half the sample for both the visual-symbol and color matching tasks (in what we called Cohort 1), we repeated the main correlation analysis between brain activation during phonological processing and handwriting skills using the participants included in this control analysis and still found significant results in the right IFGtri (*r* = −0.49, *p* = 0.024).

### VBM results

We specifically examined whether there were structural correlates of the functional finding by evaluating whether there were significant associations with the right IFG regional gray matter volume and handwriting quality controlling for total gray matter volume. We found a significant *positive* correlation between handwriting quality and regional gray matter volume in the right IFGtri, spatially overlapping with the fMRI results (TAL: *X* = 40, *Y* = 27, *Z* = 17; peak *T* = 3.66; *p* = 0.027 corrected; Figures 3B,C). The association was however, positive and in the opposite direction to the fMRI findings. Even when the whole-brain was examined rather than the a priori hypothesized ROIs, four clusters in right IFGtri—middle frontal gyrus, left IFGtri, right middle temporal gyrus, and right postcentral gyrus—intraparietal sulcus (inferior/superior parietal lobule) were the only regions that showed a significant effect at a lenient threshold of *p* = 0.001. There were no brain regions that showed significant negative association with gray matter volumes or significant positive or negative association with white matter. The positive correlation in the right IFGtri remained significant using even after regressing out variables that correlated with handwriting skills such as age, gender, visuomotor skill (rank ordered BVMI responses), BVMI (dominant/right hand) raw scores, and WJ-III Spelling raw scores (*r* = 0.536, *p* = 0.001). Further, regional gray matter volume significantly correlated with functional activation from the main analysis (*r* = −0.323, *p* = 0.043).

## Discussion

We have presented results examining beginning writers/readers' association between handwriting quality and brain activation. Our preliminary results showed that poorer handwriting quality was associated with stronger activation of the right IFGtri when children judged whether a pair of pictures starts with the same sound. Furthermore, these results overlapped spatially with reduced regional gray matter volume in the right IFGtri in children with less proficient handwriting. Brain activation during supplementary fMRI tasks, where children judged visual similarities between pairs of unfamiliar symbols and discriminated between colors, were not associated with handwriting quality. Regional gray matter volume associations were also significantly correlated with the functional associations specific to the right IFG during the phonological task. These findings show the significance of IFG in handwriting quality in beginning writers, demonstrating that increased activation in the right IFGtri during a task likely related to the phonological processes involved in reading is associated with reduced handwriting quality, which in turn showed structural brain correlates. While our control condition was rest in our phonological fMRI task because of the young age of our participants (see Methods—Functional MRI Tasks above), we believe the task taps at least partially into phonological processing. This is because other studies using comparable tasks as well as our own study have successfully shown phonological processing related reading networks to be active during the task (see Methods). Additionally, we have included two supplementary tasks to show that the findings were at least not due to more non-specific aspects of the task such as visual perception, judgment and motoric responses. The results of this study show that the neuroanatomical properties and phonologically related neurofunctional properties of the IFG may be essential in the development of complex motor skills required in handwriting.

The IFG is a heterogeneous region with many functions. Existing literature on the IFG suggests its involvement in an exhaustive list of language abilities, including: syntactic processing (Embick et al., 2000), accessing orthographic long-term memories in the form of stored motor plans (Hillis et al., 2002; Rapp and Dufor, 2011), coordinating orthographic lexical selection and retrieval (Purcell et al., 2011), verbal working memory (Paulesu et al., 1993), letter perception and letter transcription (James and Gauthier, 2006), activation during speech generation (Liotti et al., 1994), grasping and manipulating objects (Rizzolatti et al., 1988), silent naming of manipulable objects (Grafton et al., 1997), observation of manipulable objects (Grafton et al., 1997), and when handwriting novel letterforms (Longcamp et al., 2008). Regarding its purported function in relation to writing, a recent meta-analysis of handwriting studies (Planton et al., 2013) found evidence for IFG involvement in writing, and in particular when contrasted against a control motor task (e.g., vocalization), but not for contrasts that controlled for linguistic input processing. This supports the role of the IFG in processing linguistic input during writing rather than motoric output (Planton et al., 2013). In our study, we additionally show that handwriting quality correlated not only with IFG volume, but also with activation during a task that was at least partially related to phonological processing. This suggests that at the beginning stage of reading and writing, there is a tight coupling between IFG—albeit right lateralized—and handwriting, possibly via phonological processing. It is interesting to note that handwriting quality also correlated with a behavioral measure of phonological encoding (spelling). We interpret our predominant results on the right hemisphere (left hemisphere involvement was present but only at subthreshold) in terms of neuronal efficiency, which we discuss below.

Although there is evidence for IFG involvement in a variety of tasks, its robust associations with phonological processing and lexical retrieval are likely the most relevant with respect to reading. Many aspects of language processing show leftward functional asymmetry in the IFG in most adults (Price, 2010). Although children show some indication of frontal left hemisphere asymmetry, the degree of asymmetry increases into adulthood (Holland et al., 2001; Szaflarski et al., 2006). Increased left functional asymmetry for language production has been linked to increased vocabulary and non-word reading scores in children (Groen et al., 2012) and more bilateral or right hemisphere IFG activations found in disabled populations, such as reading impaired dyslexics (Calvert et al., 2000; Pugh et al., 2001; Hoeft et al., 2011). Larger activation extents in the IFG have also been reported in children during linguistic tasks (Gaillard et al., 2000; Hoeft et al., 2011). This suggests a developmental reorganization and refinement of frontal language circuits through young adulthood. Our finding of a negative correlation between children's handwriting performance and right IFG activation is consistent with a common maturational process affecting handwriting and phonological processing. Children with high activation in the right IFG during phonological processing may be developmentally delayed with respect to adult-like patterns of functional asymmetry for language processing and consequently be delayed in the development of handwriting performance, either via a direct link between phonological skills and handwriting or a more general, domain independent delay. However, the specificity of our findings argues against a general delay.

An alternative account, which does not assume functional homology between the left and right IFG, is that improved handwriting is associated with increased computational efficiency or neural coding—and hence reduced BOLD signal increase—in the right IFG for reading-related functions. This phenomenon, known as neural efficiency, posits that brighter individuals use their brains more efficiently and is often used to explain the inverse relationship between brain activation and task performance (Haier et al., 1988). A recent study by Holland et al. (2011) has shown that greater recruitment of the IFG is associated with slower naming (reduced proficiency) during a picture-naming task. Further, decrease in right IFG activation during an orthographic processing task has been shown with orthographic training, a process known to contribute uniquely to handwriting, spelling, and composition (Richards et al., 2006a). Training-induced reduction in right IFG activation has also been shown to correlate with improved phonological decoding (Richards et al., 2006b). The positive association between handwriting performance and gray matter volume may be compatible with this interpretation. Morphometric studies have found that increased regional gray matter volume may result in less energy consumption when that area is employed (Haier et al., 2004), and it is generally accepted that increased volume denotes increased cognitive capacity. This interpretation is further supported by the negative correlation between behavioral measures of phonological memory and right IFG activation during the phonological task. In our study, while both age and gender showed associations to handwriting quality (see Table 1), our findings persisted even when these factors were regressed out. Moreover, there were no significant correlations with environmental measures (e.g., Home Literacy Inventory) used as proxies to control for differential exposure to reading/writing materials. Thus, there is some indication that observed differences are not related to age or environmental differences, but instead to differences in maturational development of language related processes or neural efficiency.

Recent studies of handwriting in children have found differences in activation within the fusiform gyrus (Longcamp et al., 2008; Richards et al., 2009a,b), an area known as critical for orthographic processing and implicated both in letter and word perception, critical components for both reading development and handwriting acquisition (James and Engelhardt, 2012). Other studies note the importance of Exner's area and the SPL. Exner's area has been implicated for its role in bridging the gap between orthography and the motor programs necessary for handwriting (Roux et al., 2010; Planton et al., 2013), and the emerging consensus regarding the SPL posits that this region is involved in the abstract representation, sequential selection, and production of letter shapes (Rapp and Dufor, 2011; Planton et al., 2013; Rothlein and Rapp, 2014). We did not demonstrate a significant association between handwriting quality and neuroanatomical structure or activation in ROIs other than the IFG, such as Exner's area, fusiform gyrus and the SPL. The absence of significant results in Exner's area (though there was a trend for significance also on the right hemisphere) and the SPL may be explained by the fact that most studies that have reported these regions have used adult participants. Research has shown that in adults specific neural substrates respectively correspond to differing letter representations (Rothlein and Rapp, 2014), but this cerebral organization is likely very different in early development. It may be that the phonological processing subserved by the IFG becomes less necessary for writing as language skills become more automatic. Once this occurs, regions such as Exner's and SPL, important in the motoric and visuo-spatial component become more involved (regions thought to be specialized for fluent, automatic handwriting). It may also be the case that significant effects may have been observed in these regions if a different fMRI task was used that emphasize more motoric and visuo-spatial components, though this will not explain the lack of associations neuroanatomically. Another probable explanation is that the inverse correlation with activation in the IFG may correspond with the emergence of neural circuits in posterior writing areas in better readers. It is possible that this was not detected in our study due to the small, age-limited sample. In which case, the IFG activation may relate not to letter formation, but rather to its well-established role in motor planning and executive function. Further, while the activation observed in our study is assumed to be essential for the phonological task, some studies have shown that activation does not necessarily correspond to what is necessary for the particular tasks being administered (Rothlein and Rapp, 2014). Future studies will need to dissociate these possibilities.

The lack of association between handwriting quality and activation and neuroanatomical patterns in fusiform gyrus is more difficult to explain, especially as significant association with handwriting and reading (letter perception) was found in beginning writers / readers. While again, this requires further investigation (as described in limitations), it is possible that the lack of significant findings in the fusiform gyrus is related to the nature of the phonological task we used, as our task requires no orthographic processing, and hence no interaction was found with handwriting quality in the fusiform gyrus. Thus, it is very likely that if we included another fMRI task related to letter perception or orthographic processing as in James and Engelhardt (2012), we would have seen associations with handwriting in the fusiform gyrus (and SPL) also even though this will not account for the lack of neuroanatomical findings in this region.

Our study provides insights into why some children with dyslexia have been found to have poorer handwriting as well (Berninger et al., 2008). Previous literature has indicated children with dyslexia taught both word decoding and handwriting showed improvement in reading as well as orthographic decoding (Berninger et al., 2008; Berninger and Richards, 2011). It has also been shown in adults with pure alexia that reading performance can be improved through handwriting practice (Seki et al., 1995; Bartolomeo et al., 2002). Recently, a related study on the relationship between handwriting experience and neurological development in beginning readers showed that those with more experience printing and tracing activated the IFG during letter perception more than children with experience typing or copying (James and Engelhardt, 2012). Accepting past literature showing the IFG as important for linking features together to construct an organized whole, these researchers proposed that the IFG may be important for motor planning, control and execution. At a minimum, our study is distinguished from James and Engelhardt in that rather than investigating letter perception, our tasks did not include stimuli related to written languages (e.g., letters and words) and still found significant associations. Further, we find neuroanatomical evidence of associations between IFG and writing. Our findings hence provide novel findings adding to the important role of the process of writing in reading development.

## Future directions and limitations

Future studies investigating handwriting quality and development may assess the role of maturation, lateralization and neural efficiency related to handwriting by following children longitudinally, and by examining lower level visual and motor processing, spelling and writing compositions. Attention also plays a role in successful handwriting (McCutchen, 1996) and while we did incentivize and encourage attention, future studies may examine better ways to control or account for attention.

There are several limitations to our study that will need to be addressed in future research. First, our phonological processing task where children judged whether the initial sounds of the names of pictures matched did not have a well-matched control condition such as a picture matching condition. Although we included supplementary fMRI tasks we had available (e.g., visual matching and color matching tasks), these may have been inadequate to serve as control tasks. This determination was based on our preliminary study in young children (see Methods—Functional MRI Tasks for details). Second, while unrealistic to keep children in kindergarten in the scanner for long periods of time, future studies may include fMRI tasks specifically related to writing, orthographic, visual and motor processing in addition to phonological processing to examine task induced differences in activation patterns as it relates to handwriting. Third, while qualitative/holistic approaches remain the most common way to assess handwriting quality (Wagner et al., 2011), there is need to find more quantitative methods, such as using computer algorithms to interpret handwriting quality and errors. Fourth, the working memory condition during fMRI was not significantly different from the non-working memory condition, and hence we were unable to address the issue of working memory in writing. Fifth, the participants included in this study were gifted compared to normative populations with standardized behavioral profiles well above average (see Table 1), potentially reducing the extent to which our results are generalizable. Finally, we compared a copying task (BVMI) to a spelling task (WJ-III), and there were differences in task requirements, such as encoding differences, and letters vs. symbols, as well as other potential differences such as verbal short-term memory and visual long-term memory (remembering shapes of letters); these should be dissociated in future studies. Despite these limitations, our study is an important step in identifying the neural substrates of handwriting quality in beginning writers.

## Conclusions

In the current study, we provide evidence of direct neural links between handwriting quality, a skill that has been strongly associated with higher level writing skills and reading, and neural processing underlying phonological processing, which is thought to be causally related to reading acquisition. In contrast to studies focused on neurologically impaired individuals (e.g., Benson, 1979; Exner, 1881; Kaplan and Goodglass, 1981), we took a dimensional approach to investigate handwriting and have provided preliminary but novel evidence that the IFG may be a key link between phonological processing and handwriting quality during early phases of language development. The findings in the current study indicate that during early development of reading and writing skills, successful handwriting quality, measured by one's ability to shape and form letters coherently, relies on the right IFG, and that this efficiency corresponds to successful phonological processing.

### Conflict of interest statement

The authors declare that the research was conducted in the absence of any commercial or financial relationships that could be construed as a potential conflict of interest.
